# Needs assessment study of postgraduate surgical education in Sudan: Trainees perspective

**DOI:** 10.1371/journal.pone.0291664

**Published:** 2023-10-05

**Authors:** Elrasheid A. H. Kheirelseid, Reem Magzoub, Muhannad Ahmed, Abdulrahman A. Rudwan, Mohamed S. M. Awoda, Walter Cullen

**Affiliations:** 1 Department of Vascular Surgery, Beaumont Hospital and RCSI, Dublin, Ireland; 2 Department of Surgery, Sudan Medical Specialization Board (SMSB), Khartoum, Sudan; 3 Department of Gynaecology, Beaumont Hospital, Dublin, Ireland; 4 School of Medicine and Medical Science, University College Dublin, Dublin, Ireland; Makere University College of Health Sciences, UGANDA

## Abstract

**Introduction:**

Global interest has increased in improving the quality and increasing the number of graduates from surgical training programmes in countries with limited resources. Needs assessment of stakeholders in the training programmes represent the backbone of such process. The aim of this study was to assess the surgical training in Sudan from trainees’ perspective in order to inform training delivery.

**Methods:**

We adopted mixed methods design using focus group discussion for qualitative data collection and questionnaire survey for quantitative data. NVivo 20 Pro was used to organize qualitative data and SPSS 24.0 was used for quantitative data analysis.

**Results:**

Thematic analysis of qualitative data identified three themes. Trainees were overall satisfied that they will make good surgeons after completion of the programme. They identified case volume and collaborations with colleagues as the main strengths of the programme and lacking clear objectives for each year of training and academic activities as the main weaknesses. They suggested motivation of trainers and utilization of online resources and meeting platforms as solutions to improve supervision and academic activities during training.

**Conclusion:**

The gaps in training and their suggested solutions highlighted by trainees in this study should form the base for reforming the surgical training in Sudan and countries with similar circumstances.

## Introduction

Increased burden of surgical disease with limited accessibility to surgical care in countries with low resources has become a global concern [1, 2]. That resulted in over two billion people lacking access to basic surgical service with an estimated 1.5 million deaths per year [3, 4]. Limited accessibility to surgical care in these regions is caused by numerous factors including severe shortage of trained surgeons [2]. Like other countries in the region, interest in Sudan has increased on how to train healthcare professionals with adequate surgical skills to meet the demand for surgeons in the country.

Although Sudan, with estimated population of over 46 million is the third largest country in Africa, [5] physician density in the country is among the lowest in the world with most medical practitioners located in the big cities [6, 7]. Despite the fact that medical and health services in Sudan were established in 1898, training of Sudanese doctors only started in 1924 and specialized surgery service was not developed till 1949 [8]. Since it was established in 1970s, the surgical training in the Sudan has gone through numerous phases of reforming. Historically, Sudanese surgeon had to go abroad, namely to the UK, for surgical training and certification. The national postgraduate training in surgery was then established by the postgraduate colleges of the universities, mainly the university of Khartoum. Thereafter a more successful collaborations were established with United kingdom and Ireland [9]. Currently, the surgical training is regulated and delivered by Sudan Medical specialization board (SMSB) which was established in 1995. By the year 2000, SMSB was the only authority to provide surgical training in the country. It awards clinical MD in general surgery and orthopaedics and fellowship in other surgical subspecialties Despite the confirmed role of collaborative partnership and online resources and e-learning in improving the quality of surgical training in low-income countries, their effect could not be achieved if they do not address the needs of key stakeholders. Therefore, surgical training in Sudan should be systematically assessed and evaluated in order to improve the quality and volume of the training in the country to match the global advances in surgical practice.

Needs assessment, defined as the process of identifying performance requirements or gaps between current and desire performance, should always be the initial step in developing and refining learning and training activities as indicated in adult learning theory [10, 11]. Trainees’ opinions regarding their educational needs does not always match their trainers or other stakeholders [12, 13]. Therefore, they should be assessed separately and in connection with needs identified by other learning methods and stakeholders to achieving a successful training process. Although some reports are available from countries with low resources, still there are gaps in the literatures, especially about trainees’ opinion on how to solve the problems in their training programme. In addition, currently no reports are available specific to surgical training in Sudan to address this issue. Therefore, we carried out this study to characterise and evaluate surgical training in Sudan by performing needs assessment from the surgical trainees’ perspectives. The outcome of this study should help in assessing the role of previously identified interventions from similar regions to improving training quality and ultimately lead to systematic improvements in surgical education.

## Materials and methods

### Study design

The study was multi-phase, mixed methods [14] cross-sectional study conducted between January and June 2022. Sequential exploratory design was adopted in which we firstly collected qualitative data using focus group discussions (FGD) as an exploratory phase. That was followed by quantitative data collection with a larger sample using survey questionnaire to generalize and explain findings. This study was designed to ensure that rigour, fitness for purpose and high quality of data collection, analysis and reporting are addressed. Attention was made during planning to overcome the problems associated with mixed methods including representation, legitimation and integration [15]. Participants were sampled from the same population for both qualitative and quantitative studies to ensure sample integrity. In addition, data triangulations was displayed through using more than one method for data collection and data from different people from different locations at different time was gathered [16].

Study protocol was approved by the SMSB Research Ethics Committee. Participation in the study was voluntary and an informed consent was obtained from each trainee before participating in focus group discussion (FGD) or completing the study questionnaire. The consent process for participating in the study was also approved by the ethics committee of SMSB. For the FGD, the consent process was included in the FGD protocol. That consist of sending leaflet with overview description of the study at the time of invitation to participate in the study. On the day of the FGD, the description of the study and the FGD were explained to participants and then informed verbal consent was obtained before being included in the group discussion. The consent process was witnessed by other participants and kept as a separate audio file. For the participants in the survey, informed written consent was obtained at the start of the questionnaire. Signing the consent was mandatory before being able to submit the questionnaire. Anonymity, confidentiality and right to withdraw were emphasised to all participants.

### Study population

Surgical training in Sudan is solely regulated and delivered by the Sudan Medical Specialization Board (SMSB), which was established in 1995. It offers a five-year training programme in general surgery. Selection process for the programme depends on passing part 1 SMSB surgery examination, after which trainees will get enrolled in the two-year core training programme. The core training programme consists of two 6-month rotations in general surgery and three four-month minor shifts in other surgical specialties. After completing the core training and passing the exam trainees will the get promoted to the three-year speciality training in general surgery, plastic surgery, urology or paediatrics surgery. After successful completion of the five years training period, submitting research thesis and passing the final exit examination, trainees will be awarded MD in surgery. At the time of the study, there were about 600 trainees registered in the programme.

### Qualitative data

Initially, qualitative data was collected using focus group discussion (FGD). Invitation to participate in the FGD was sent to fifty randomly selected trainees from the trainee’s email list. Off them, 25 replied to the email willing to participate in the study. Seven trainees did not show up on the day of the FGD due to work commitment or poor internet connection. The FGD data reached saturation after round 4.

Seventeen trainees participated in the FGD four rounds with average of 4–5 participants per group. Protocol for the FGD was developed to include a list of research questions and interview guide. All FGDs were conducted by video group call and recorded using Zoom platform (Zoom video communications, Inc., San Jose, California, U.S.). They lasted between 50 and 90 minutes.

Qualitative data was analysed using inductive thematic method adopting the framework described by Braun and Clarke [17]. Transcribed data was imported into NVivo 20 Pro for Windows and Mac (QSR International Pty Ltd, Australia) to organise and support the coding process. Codes were generated by reading the transcripts one ward at a time and then categorised into subthemes by combining similar codes. Final themes then revealed. Codes refining and themes evaluation was then reassessed manually and through discussion with supervisor.

### Quantitative data

A structured 37-item questionnaire (S1 Table) was developed for the purpose of this study based on literature review of similar studies of needs assessment from trainees’ perspectives that were conducted in similar settings and on the results of the qualitative exploratory data from the focus group discussion. The data collected include participants demographics, trainees views on the training programme and available resources and their suggestions for improving the quality of training, using multiple choices and five points Likert-scale questions The questionnaire was piloted with 25 trainees to assess the clarity of questions and rephrased accordingly before it was distributed to participants. In addition, the reliability and validity of the questionnaire was assessed and the internal consistency was measured employing Cronbach alpha coefficient which was 0.883.

The study questionnaire was distributed electronically via SurveyMonkey ^TM^ to the 600 trainees currently registered in the SMSB surgical training programme. A survey reminder was sent to potential participants every two weeks for a total of four attempts before closing the survey after 8 weeks. By the end of recruitment period only 130 trainees of the 600 contacted accessed and opened he questionnaire through SurveyMonkey ^TM^.

Both complete and incomplete questionnaires were included in the analysis. Statistical Package for Social Sciences 24.0 for Windows (SPSS Inc., IBM, New York, USA) was used for data analysis. Percentages, frequencies and means ± standard deviation (SD) were used for descriptive statistics. Inferential statistics to compare differences between the groups (e.g., speciality, stage of training, sex, age) was performed using Chi-squired and Fischer exact test for categorical data and Mann-Whitney U, student-t test and ANOVA for numerical data. Statistical significance is accepted at p ≤ 0.05, and all tests are two-sided.

## Results

### Qualitative data

**Fig 1:**
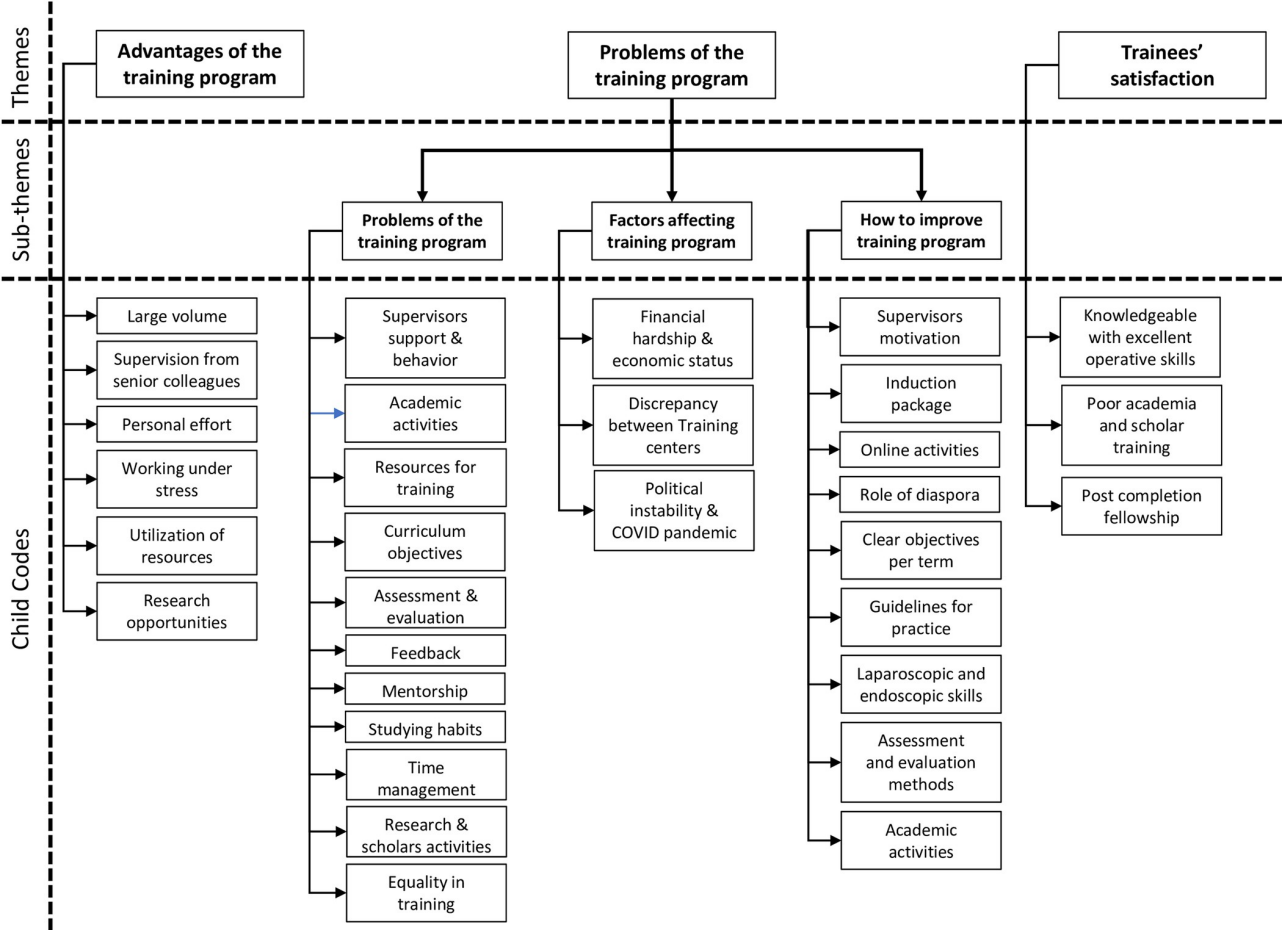
Thematic analysis outcome. Explore diagram to show themes, sub-themes and child codes revelled by trainees in the study.

Seventeen trainees participated in four rounds of focus group discussion (FGD). Eleven of the participants were females and 6 were males. Two of the participants were in 5^th^ year of training (R5), one was R1, three were R2, eight were R3 and three were R4. Thematic analysis of the qualitative data revealed three themes: Advantages of the training programme, Problems of the training programme and overall satisfaction about the programme (Fig 1).

### Advantages of the programme

Cases volume and pathology mix and support from senior trainees colleagues were described by the trainees as the main strengths of training in surgery in Sudan. Due to the tough working environment and limitation of resources, in addition to, the lack of support from trainers specially for emergencies, trainees developed skills that they considered as advantages of the training programme. These advantages included building self-confidence, ability to work under stress and effective utilization of the limited resources.

### Problems of the training programme

Participants in the FGDs spent considerable amount of time discussing the problems of their training programme. In addition, they tried to analyse the factors contributing to these problems and to giving suggestions for solutions in order to improve the quality of training.

Participants described unavailability of consultant trainer, time pressure, lacking of clear objectives for each year of training and insufficient academic activities as the major problems of training in surgery in Sudan. Other problem reported included limited feedback provision and evaluation and assessment timing and tools.

Trainees highlighted the political instability in Sudan, financial hardship and the economic status and COVID pandemic as the main causes of problems in training in surgery. Political instability and covid pandemic, in addition to their effect on economy and healthcare service in general, they caused shift in cases volume from public to private hospitals. That is due to understaffing and lack of resources for investigations and management of patients at public hospitals. Furthermore, financial hardship made both trainers and trainees to look for financial support by working extra-time at private hospitals. Therefore, trainer were not readily available at training centres and trainees could not find time for studying or to perform academic or scholar activities. Participants suggested financial support for trainees by providing regular salary and also financial incentive, courses and scholarships to motivate trainers and make them more interested in the training process.

Another factor that is contributing to the problems in training from participants point of view is the discrepancy between the training centres, especially when comparing centres in Khartoum to centres outside Khartoum. Trainees stated that Khartoum centres are good for academic activity, improving knowledge and preparing them for examinations, while centres outside Khartoum are better to learn operative and clinical skills. They attributed that to the availability of trainers in public hospitals and also the limited number of private hospitals outside Khartoum.

To improve the academic activities quantity and quality and also research producibility, participants suggested better utilization of virtual platforms and online resources and also involvement of the diaspora in teaching, mentorship and research supervision. They also highlighted the importance of communicating the training objectives for each year of training to the trainees and to evaluate and assess them against these objectives with timed feedback provision. In addition, trainees reported the need for induction package at the start of the programme.

### Overall satisfaction of trainees

Despite the reported problems facing trainees during the training process; overall, the participants confirmed that they are satisfied that they will make excellent surgeons and clinicians after completion of their training. Nevertheless, they were concerned about their teaching and research skills. Most of trainees stated that they will look for fellowship position abroad before taking a consultant post and commit themselves to training other doctors. Quotations by participants from the focus group discussion rounds were demonstrated in Table 1.

**Table 1:** Quotations from focus group discussion rounds.

Theme	Participant characteristics	Quotation
**Advantages of the programme**	Male, R5, FGDR2	*“The main strength of the surgical training programme in Sudan is the operative skills and also the confidence you build during the training working under great stress”*
	Female, R2, FGDR1	*“I think our difficult exams also helped us to having excellent basic science theoretical knowledge”*
	Female, R3, FGDR1	*“In my opinion the good thing about training is Sudan is the large volume of surgical cases the varieties in pathologies”*
**Problems of the training programme**	Female, R3, FGDR1	“*Me personally don’t usually get feedback or supervisor advices about progress or how I am performing*. *But feedback in relation to surgical skills is good*”
	Male, R2, FGDR1	*“I am at the second year of training and I am not satisfied about my training progress*. *May be the pandemic lockdown has an effect*, *but the number of patients attending public hospitals with surgical problem small and most of patients go to private hospitals*. *That created competition between trainees for operative theatre activities”*
	Male, R5, FGDR2	“*The main problem with training in Sudan is related to the poor economic status in the country which is reflected in all healthcare service*”
	Male, R5, FGDR4	“*To improve the quality of the programme there should be clear objectives for each year of training and that will remove significant stress from trainees*. *It also needs more organization*”
	Female, R3, FGDR4	“*Sudanese doctors working abroad can help by organizing online activities and workshops*, *but there is the problem of availability of internet*. *Also they can help by supervising research projects and help us in writing papers especially with available platforms that made virtual communication easy”*
	Female, R4, FGDR4	*“Trainers assessment and evaluation has to be implemented which should be feedback to trainers to help in improving quality of training and availability of trainers”*
	Female, R3, FGDR1	*“I think the quality of training is better in centres outside Khartoum compared to Khartoum centres as there is higher number of cases and also consultant trainers are available at all times”*
	Female, R3, FGDR2	“*Problems with infrastructures*, *equipment and staffing in public hospitals made training difficult as patients now preferer to be treated in private rather than public hospitals”*
**Overall satisfaction of trainees**	Male, R5, FGDR4	*“The training programme prepares you to be a good consultant but requires significant effort from trainees”*
	Male, R5, FGDR3	*“I think I will be a good consultant after completing this programme as I built adequate knowledge and skills during my training*, *but I will be lacking research and scholar skills*. *The programme could help in achieving such lacking skills but needs better organization and structure”*
	Female, R3, FGDR4	*“I don’t think I can work as a n independent consultant after finishing this programme and I will still need to seek fellowship abroad before taking the responsibilities of a consultant and trainer”*

***FGDR***: Focus Group Discussion Round, ***R***: year in training as registrar

### Quantitative data

The total questionnaire response rate was 21.3% for all surveys electronically distributed (128 of 600) and 98.5% for all surveys distributed and opened (128 of 130). All survey data is available in S1 File. Most of the respondents were males (66.4%) and 43.8% of them were between 30–34 years of age. Demographic details are shown in Table 2 and the questionnaire items Likert score means are demonstrated in Table 3. No significant statistical difference was observed comparing any of the questionnaire items Likert score means based on gender, training years or age groups.

**Table 2:** Demographic details of survey participants.

Category		Frequency (n = 128)	Percentage
**Gender**	Male	85	66.4%
	Female	43	33.6%
**Year of training**	R1	26	20.5%
	R2	20	15.7%
	R3	33	26%
	R4	14	11%
	R5	34	26.8%
**Age group (Years)**	20–24	2	1.6%
	25–29	44	34.4%
	30–34	56	43.8%
	35–40	23	18%
	Older than 40	3	2.3%

**Table 3:** Questionnaire items Likert score means.

Questionnaire item	Likert scale Mean	SD
I have a contract of employment that provides information about hours of work	1.97	1.10
I have informative induction programme.	1.86	0.35
My progress is tracked and my trainers provide me with good feedback about my strengths and weaknesses.	2.67	1.15
I have to perform inappropriate tasks	1.34	0.48
The programme has clear training goals for each year of training.	1.98	0.99
There is informative curriculum handbook	1.86	0.35
The curriculum prepares me adequately for my final exams.	2.03	0.90
My trainers usually set clear expectations.	1.73	0.44
I have protected educational time during my training.	1.85	0.36
I have good clinical supervision at all times	2.36	1.12
My trainers encourage me to be an independent learner	1.28	0.45
I get evaluation by SMSB at the end of each year with clear feedback on my performance over last year and plan for next year.	1.82	0.83
There is sex discrimination in my training programme	1.56	0.50
I have good collaboration with other doctors in the same year of training.	3.7	1.19
I feel physically safe within hospital environment	1.89	0.38
My trainers have good mentoring skills	1.42	0.50
My programme has fear-free environment so that I can report problems without fear of reprisal.	2.30	1.10
I have access to electronic databases	1.80	0.40
The amount of operative experience as a first assistant	3.46	1.07
The amount of operative experience as the primary surgeon	3.17	1.10
The amount of supervision in the operating room by training consultant	3.07	1.14
The amount of supervision in the operating room by senior trainee	3.26	1.08
The opportunity I get to develop my leadership skills	2.98	1.15
The amount of time to study	1.94	0.95
The opportunity to present cases in rounds	2.91	1.01
The opportunity to see ambulatory patients in a clinic setting	3.36	1.06
The amount of training in ethical issues including how to communicate properly with patients	2.73	1.19
I have adequate access to endoscopy training	1.63	0.90
I have adequate access to laparoscopy training	1.71	0.96
My access to books and journals	2.11	0.97
My access to information on the Internet	3.01	1.13
My access to a surgical skills laboratory (eg. operating on cadavers, models etc)	1.48	0.79
The amount of time I work per week (regular + on call)	2.60	1.29
My opportunity to pursue clinical and/or laboratory research activities	1.96	0.93
When I complete my training, I will feel competent to practice as a consultant	2.93	1.06

### Training facilities and resources

**Fig 2:**
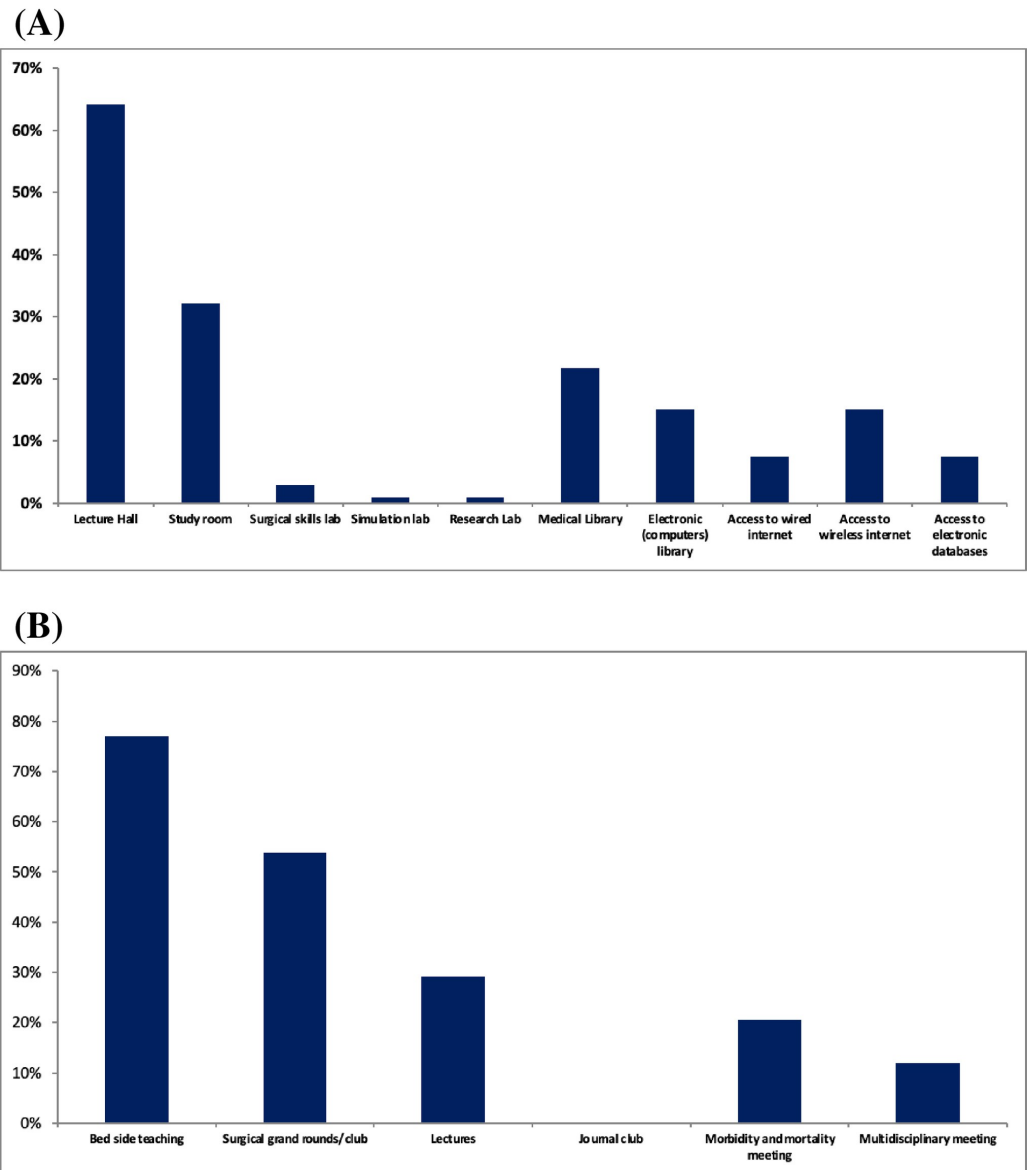
Availability of (A) training facilities and (B) training activities for trainees in Sudan.

The most available facilities that were reported by trainees were lecture halls (64.2%), study rooms (32.1%) and medical library (21.7%), in order. In addition, electronic library was available for 15.1% of the trainees with access to electronic databases reported by only 7.6%. Moreover, there was a very limited access to simulation and research laboratories (less than 1%) and surgical skills laboratory (2.8%) (Fig 2). The participants appreciated that they have adequate access to information on the internet (mean score: 3.01±1.13) and to books and journals (mean score: 2.11±0.97).

### Academic activities

Bed side teaching (77%) and surgical grand rounds (53.9%) were the most available teaching activities available for the trainees that were reported by the participants. However, journal club was not available for any of the trainees during their training (Fig 2). Most of trainees were satisfied about the opportunity to review patients at ambulatory clinic setting and to present at grand rounds with mean Likert score of 3.36±1.06 and 2.91 ± 1.01, respectively.

### Research training and support

Opportunity to perform clinical research was reported by most of the trainees (71.6%). However, they were largely dissatisfied with their experience performing clinical or laboratory based research (mean score: 1.96±0.93). In addition, they described limited mentorship (17.1%) and availability of research grant (15.9%). Only 1% of respondents had the opportunity to participate in laboratory based research.

### Curriculum

In examining curriculum components participants agreed that the current curriculum does not prepares them adequately for their final exams (mean score: 2.03±0.90). In addition, they perceived that the programme does not have clear objectives for each training year (mean score: 1.98±0.99). Nevertheless, the mean Likert scale response was more than 2 for the trainees opinion on their ability to develop leadership skills and training in ethics and communications with patients during their training (2.98±1.15 and 2.73±1.19 respectively). Looking at the evaluation process by SMSB, trainees reported low satisfaction rate with evaluation at the end of the year of training, the feedback provided on their previous year performance and the plan for next year (mean score 1.98±0.99).

### Learning environment

Some items included in the survey assessed the perception of role autonomy, teaching and social support during training (Table 4). All items examined had mean score more than 2, except for Mentoring skills of trainers, physical safety within hospital and availability of informative induction programme for trainees.

**Table 4:** Learning environment questionnaire items.

Perception	Item	Mean (SD)
**Teaching**	Clinical supervision	2.36 (1.12)
	Feedback from trainer	2.67 (1.15)
**Social support**	Fear-free training environment	2.30 (1.10)
	Collaboration with peers	3.73 (1.19)
	Mentoring skills of trainers	1.42 (0.50)
	Physical safety with hospital	1.89 (0.38)
**Role autonomy**	Informative induction programme	1.86 (0.35)

### Operative skills

Trainees included in the survey believe that they experience appropriate amount of training as first assistant and primary surgeon with mean score of 3.46±1.07 and 3.17±1.10, respectively. Furthermore, they were satisfied with the amount of supervision they receive from their trainers (mean score: 3.07±1.14) and from their senior peers (mean score: 3.26±1.08). However, they perceived limited exposure to laparoscopy (1.71±0.96) and endoscopy (1.63±0.90) training and to get training in simulation and skill laboratory (1.48±0.79).

### Overall satisfaction

Although trainees satisfaction was low regarding availability of time for studying and performing academic activities and research (1.93±0.95), it was acceptable regarding workload (2.60±1.29) and their ability to practice as consultants after completion of training (2.93±1.06).

### Strengths and weaknesses of the programme

**Fig 3:**
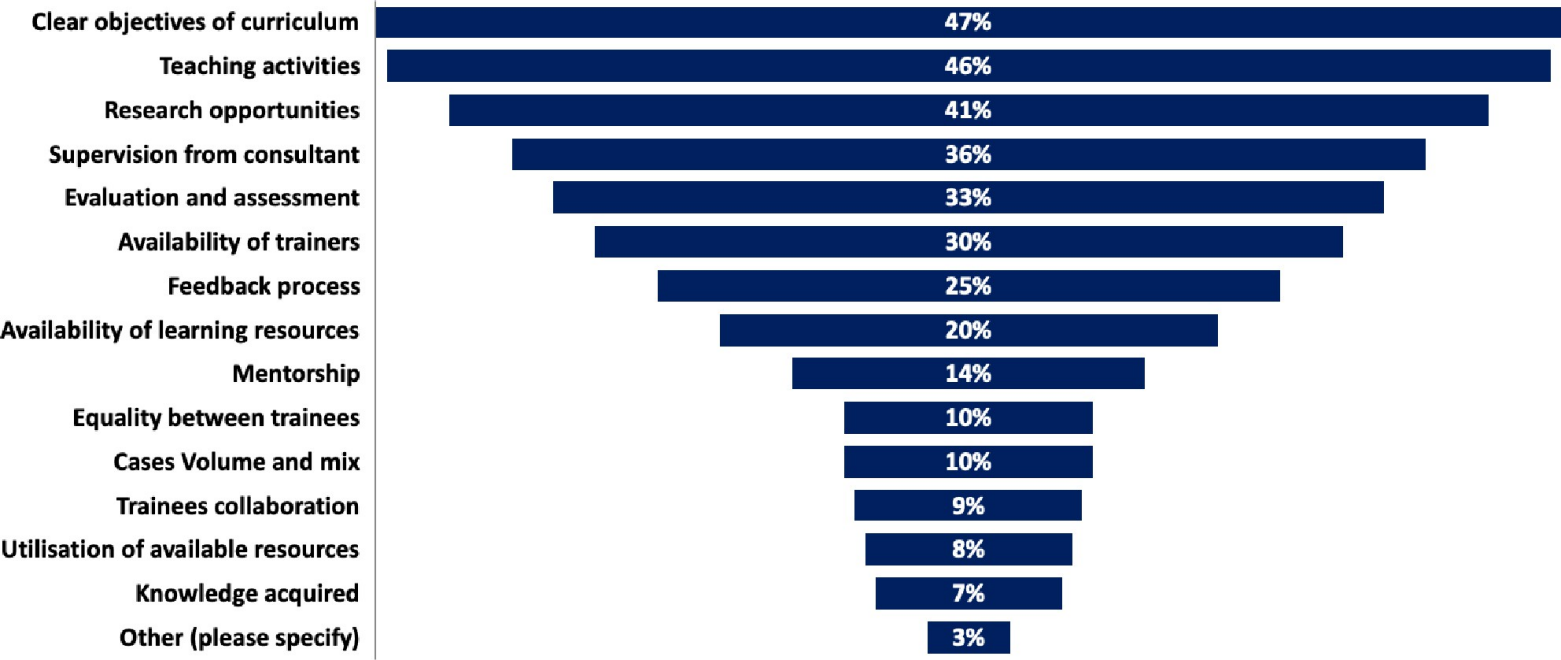
Areas that need to be improved in the surgical training programme.

Participants were asked to select the top three strengths and weaknesses of the programme in their opinion and to highlight the areas that needs further improvement to augment the quality of training. The strengths of the programme were felt to be the large volume and mix of the surgical cases (68%), the collaboration between trainees (43.7%) and the support from trainers (28.6%). On the other hand, weaknesses highlighted by trainees included lack of clear objectives for each year of training, (45.8%) and limited research opportunities (45%) and teaching activities (40%). To improve the quality of training, trainees advised more attention for each training year goals (47%) teaching activities (46%) and research training and support (41.3%) (Fig 3).

## Discussion

Reforming of surgical training is in demand in Sudan to improve the quality of training and the number of trainees. Needs assessment findings from trainees perspective vary, depending on region of training and availability of recourses. Although some reports are available from countries with similar circumstances, addressing the needs specific to Sudan is mandatory. We adopted mixed method design as it gives a richer and more reliable understanding of the research question [18]. In addition, it is claimed to enable more comprehensive and complete understanding of the topic investigated and answers research questions more meaningfully as methods complement and supplement each other [14, 19]. Moreover, it can increase the accuracy of the data and the reliability and enables compensation between strength and weaknesses of research methodology and strategies [16].

To our knowledge, this is the first study to use mixed methods to assess training needs from surgical trainees’ perspectives from a country with limited resources. We identified gaps that the surgical trainees described in their training programme, which can provide guidance for improving surgical education in Sudan and in countries with similar circumstances. Participants described cases volume and mix of pathologies in addition to the support from senior trainees as the main advantages of training in Sudan. They also stated that working under pressure helped them to build confidence and develop sound leadership skills. Overall, they were satisfied with their training and confident that they will make good surgeons after completion of training. Nevertheless, they identified some problems with the training programme. In the trainees’ opinion, the problems in training are mainly related to the political instability of Sudan and the economic status of the country. They also think that COVID 19 pandemic might have some effect specially in diverting patients from public to private hospitals.

Limited academic activities were described as the main problem with training in this study. Unsurprisingly, some academic facilities like surgical skills lab, simulation centres and research lab were not available for trainees to compensate for shortage in trainers and economic tightness [20]. Furthermore, academic activities like journal club were very limited despite its confirmed role in keeping practice up-to-date and promotion of learning [21]. The second major problem with training in Sudan is the availability of training supervisors and supervision is mainly provided by senior registrar colleaques. Trainees related the unavailability of their trainers to the financial hardship and the competition in the private hospital which require more frequent presence of the surgeon in the hospital than previously needed. The suggested motivation of supervisors financially or by organising courses or scholarships for them. Furthermore, lacking of clear objectives for each year of training and effective induction programme were also highlighted as areas need attention in the programme. Trainees advised induction handbook and clear objectives that they would be assessed and evaluated against at the end of each training shift. They also described defective feedback provision and timing.

Operative volume is a key aspect of surgical training. Despite the satisfaction of the participants with the cases volume and their opportunity to operate as first assistant or primary surgeon, participants were looking for more exposure to minimal invasive surgery and endoscopy. They also described the importance of simulation centre in building confidence in acquiring such skills as they could be used repeatedly by multiple trainers with low cost. Regarding research, trainees in Sudan have the opportunity to conduct research as a mandatory requirement for successful completion of the training programme. However, they were dissatisfied with the experience due to lacking mentorship, resources, time and research grant. They also have very limited opportunity to perform laboratory-based research. Therefore, they do not usually get engaged in any research projects other than what is required for graduation. As they appreciate the importance of research for evidence based practice and contentious professional development, our trainees suggested utilization of virtual platforms and diaspora with experience in research to improve the productivity and the quality of research. Furthermore, the programme learning environment was acceptable regarding teaching and social perception with dissatisfactory role of autonomy indicated by lacking of contract and induction programme at the start of the rotation.

Few studies were identified that investigated educational needs from surgical trainees point of view in limited resources regions (LRRs) in a systematic manor. Their findings were in keeping with this study regarding the reported problems of the training programme. Available literature indicated that trainees from LRRs believe that they perform high volume of surgical operations but with minimum supervision and less exposure to specialized procedures [22, 23]. They also highlighted dissatisfaction of trainees regarding acquired cognitive and affective skills during their training [22]. In addition, study from Zambia showed lacking of surgical skills simulators and research laboratory with limited access to research mentorship, basic science and grant application guidance [23]. Furthermore, a report by the South Africa society of surgeons in training reported that trainees were dissatisfied with the amount of formal academic teaching, level of supervision in operative theatre and their exposure to minimally invasive surgery [24]. Moreover, a report from ophthalmology training in Nigeria suggested more resource materials and cases volume for further improvement in surgical and diagnostic skills [25].

A major limitation of this study is the low response rate of all surveys distributed which is a well-known problem with electronic surveys in general [26, 27]. Specific to this study, low response rate might be related to cost and availability of internet. To overcome that we persevered on making the questionnaire short and easy to fill. Methods previously described to improve response rate includes limiting distribution to known individuals, increase frequency of reminders or offering financial incentive. We avoided limiting distribution to known trainees as it might create bias. In addition, our limited resources made it difficult to motivate participants financially. However, we were able to work on improving response by advertising the study on the SMSB web page and also by increasing the frequency of reminders. To avoid limitations related to mixed methods, this study was designed to ensure that rigour, fitness for purpose and high quality of data collection, analysis and reporting are addressed. Attention was made during planning to overcome the problems associated with mixed methods including representation, legitimation and integration [15].

## Conclusion

Carrying out mixed method study design, we investigated the needs to improve the quality of the surgical training in Sudan. The problems revealed in this study and the suggestions for their solutions could form the base for reforming the training programme to meet the global requirement for surgical practice in Sudan and other countries with similar situation.

## Supporting information

S1 Table Survey questionnaire. The questionnaire items used for collecting qualitative data for the study.
(XLSX)

S1 File Questionnaire data. The excel file contain all data that was collected using the survey questionnaire.
(DOCX)
