# Supplementary material for: Needs assessment study of postgraduate surgical education in Sudan: Trainees perspective
Source: PLoS One. 2023 Oct 5;18(10):e0291664. doi: 10.1371/journal.pone.0291664 (PMC10553270; doi:10.1371/journal.pone.0291664)
Supplement: S1 File — The excel file contain all data that was collected using the survey questionnaire. (DOCX) [file pone.0291664.s002.docx]

- What is your gender?

Male

Female

- What year of training are you at?

R1

R2

R3

R4

R5

- What is your surgical speciality?

General surgery

Plastic surgery

Paediatric Surgery

Urology

Other

- What is your age group?

20 – 30

30 – 35

35-40

older than 40

- Which of the following facilities available to you as a surgical trainee?

Lecture Hall

Study room

Surgical skills lab

Simulation lab

Research Lab

Medical Library

Electronic (computers) library

Access to wired internet

Access to wireless internet

Access to electronic databases

- Which of the following teaching activities available to you as a surgical trainee?

Bed side teaching

Surgical grand rounds/club

Lectures

Journal club

Morbidity and mortality meeting

Multidisciplinary meeting

- Which of the following research opportunities and resources available to you as a surgical trainee?

Opportunity to participate in clinical research

Opportunity to participate in lab research

Mentorship in research

Grant for research

***For the following questions please choose the number that best reflects your score***

***(where 1 = Very dissatisfied, 2 = Dissatisfied, 3 = Neither satisfied nor dissatisfied, 4 = Satisfied, 5 = Very satisfied)***

- I have a contract of employment that provides information about hours of work
- I have informative induction programme.
- My progress is tracked and my trainers provide me with good feedback about my strengths and weaknesses.
- I have to perform inappropriate tasks
- The programme has clear training goals for each year of training.
- There is informative curriculum handbook
- The curriculum prepares me adequately for my final exams.
- My trainers usually set clear expectations.
- I have protected educational time during my training.
- I have good clinical supervision at all times
- My trainers encourage me to be an independent learner
- I get evaluation by SMSB at the end of each year with clear feedback on my performance over last year and plan for next year.
- There is sex discrimination in my training programme
- I have good collaboration with other doctors in the same year of training.
- I feel physically safe within hospital environment
- My trainers have good mentoring skills
- My programme has fear-free environment so that I can report problems without fear of reprisal.
- I have access to electronic databases
- The amount of operative experience as a first assistant
- The amount of operative experience as the primary surgeon
- The amount of supervision in the operating room by training consultant
- The amount of supervision in the operating room by senior trainee
- The opportunity I get to develop my leadership skills
- The amount of time to study
- The opportunity to present cases in rounds
- The opportunity to see ambulatory patients in a clinic setting
- The amount of training in ethical issues including how to communicate properly with patients
- I have adequate access to endoscopy training
- I have adequate access to laparoscopy training
- My access to books and journals
- My access to information on the Internet
- My access to a surgical skills laboratory (eg. operating on cadavers, models etc)
- The amount of time I work per week (regular + on call)
- My opportunity to pursue clinical and/or laboratory research activities
- When I complete my training I will feel competent to practice as a consultant
- The best three strengths of your training programme are: (please select three options only)

Cases Volume and mix

Teaching activities

Research opportunities

Availability of trainers

Supervision from consultant

Trainees collaboration

Evaluation and assessment

Feedback process

Mentorship

Clear objectives of curriculum

Availability of learning resources

Utilization of available resources

Knowledge acquired

Equality between trainees

Others (please specify)

- The three biggest weaknesses of your training programme are: (Please select  three options only)

Cases Volume and mix

Teaching activities

Research opportunities

Availability of trainers

Supervision from consultant

Trainees collaboration

Evaluation and assessment

Feedback process

Mentorship

Clear objectives of curriculum

Availability of learning resources

Utilization of available resources

Knowledge acquired

Equality between trainees

Others (please specify)

- What would like to see improved in your training programme? (Please select three options only)

Cases Volume and mix

Teaching activities

Research opportunities

Availability of trainers

Supervision from consultant

Trainees collaboration

Evaluation and assessment

Feedback process

Mentorship

Clear objectives of curriculum

Availability of learning resources

Utilization of available resources

Knowledge acquired

Equality between trainees

Others (please specify)
